# Dietary intake of women with and without eating disorder history from pregnancy to 3 years postpartum: a longitudinal analysis

**DOI:** 10.1007/s40519-026-01841-9

**Published:** 2026-03-27

**Authors:** Louise Auguste Schmidt, Jana Katharina Throm, Annica Franziska Dörsam, Katrin Elisabeth Giel

**Affiliations:** 1 Department of Psychosomatic Medicine and Psychotherapy, University Hospital Tuebingen, Osianderstrasse 5, 72076 Tuebingen, Germany; 2 Center of Excellence for Eating Disorders (KOMET), University Hospital Tuebingen, Tuebingen, Germany

**Keywords:** Eating disorders, Eating behaviour, Nutrition, Pregnancy, Postpartum

## Abstract

**Background:**

Pregnancy and the postpartum period are vulnerable phases, particularly for women with eating disorder (ED) history. While prior nutritional research has mainly focused on pregnancy, little is known about long-term dietary patterns in this population. This study examined dietary intake in women with and without ED history from pregnancy to 3 years postpartum.

**Methods:**

A validated food frequency questionnaire (FFQ) assessed dietary intake in women with ED history (ED group, T1: active ED: *n *=  12, past ED: *n *= 12) and healthy controls (HC group, T1: *n *= 33). Dietary patterns, food groups, as well as macro- und micronutrient intakes were analysed during pregnancy (T1), 1 year postpartum (T2), and 3 years postpartum (T3).

**Results:**

Omnivorous diets predominated (T1: 75.4%, T2: 73.2% T3: 74.4%), with no significant differences between groups or over time. However, women with ED history consumed fewer animal-based, energy-dense, sugar- and fat-rich foods. Nutrient intake during pregnancy was comparable between groups. Postpartum, the ED group showed lower fat intake (*p *= 0.027, T2) higher fiber intake (*p *= 0.037, T3) and lower vitamin B12 intake (*p *= 0.031, T3).

**Conclusion:**

Nutrient supply during pregnancy was comparable between groups, which is encouraging. Differences in food group consumption may reflect a greater health consciousness or residual restrictive behaviors in women with ED history. Given the known increase in ED symptoms after childbirth, these findings emphasize the need to further examine this nutritionally and psychosocially vulnerable period.

Level of evidence: Level 3.

## Introduction

The health of mother and child during and after pregnancy may particularly be affected by disruptive factors such as eating disorders (EDs) [1, 2]. The importance of the mother’s nutritional status for embryonic and fetal development is clearly established [3, 4] and underscored by the concept of early metabolic programming [5]. Maternal EDs, including Anorexia Nervosa (AN), Bulimia Nervosa (BN), Binge Eating Disorder (BED), and Other Specified Feeding or Eating Disorders (OSFED), may have a significant impact on pregnancy, delivery, birth outcomes as well as on fetal development due to symptomatic disturbed eating behavior and body weight [6–8].

A recent meta-analysis estimates the prevalence of EDs during pregnancy between 0.5 and 10.6%, with a mean of 4.3% [9]. It is assumed that a subgroup of women with ED history pay particular attention to improving the quality of their diet during pregnancy to promote optimal child development [10] and often report more comprehensive nutritional knowledge as compared to healthy controls [10–12]. Concurrently, the presence of ED-related symptoms may persist [13], and weight concerns might increase during pregnancy [14] or worsen postpartum [15, 16].

Nevertheless, only a few studies have examined dietary intake during pregnancy in women with ED history, and none of them report data beyond childbirth [1, 2, 17, 18]. While Nguyen et al. [17] report a higher diet quality score in women with ED history, Micali et al. [1] identified distinct dietary patterns and food groups in women with ED history. Specifically, women with lifetime EDs scored higher on ‘vegetarian’ dietary patterns, while those with lifetime AN + BN showed higher scorings on both ‘health-conscious’ and ‘traditional’ dietary patterns. Siega-Riz et al. [18] assessed differences in food group consumption, as well as in macro- and micronutrient intakes, among women with BED before and during pregnancy. Their findings included a higher consumption of fats, sweets, and coffee, displayed by a higher total energy intake.

A better understanding of dietary intake is crucial for evaluating long-term effects of EDs on maternal and child health, underscoring the importance of longitudinal assessments of dietary patterns, food group consumption and macro- and micronutrient intake [2, 17]. The present study therefore not only examined dietary intake during pregnancy but extended observations into the postpartum period, capturing the vulnerable postpartum period in women with ED history compared to healthy controls.

Based on the theoretical background and previous evidence suggesting distinct dietary behaviors in women with EDs, we hypothesized that women with a history of EDs would show greater adherence to alternative dietary patterns (e.g., vegetarian diets), more restrictive patterns in food group consumption, and a higher likelihood of deviating from national macro- and micronutrient intake recommendations during and after pregnancy compared to women without an ED history.

## Methods

### Participants

The data used in this substudy were obtained from the EMKIE study, a longitudinal family cohort study conducted by the Translational Psychotherapy Research Section at the University Hospital Tuebingen. Fifty-seven women participated in this study, including 24 women with a past or current ED (ED group) and 33 women without EDs (HC group). They were recruited between 2018 and 2022 and observed at four time points from pregnancy up to 3 years postpartum. At study inclusion, the diagnosis of a past or current ED was assessed using the German version of the Eating Disorder Examination Interview (EDE-I) [19, 20]. Throughout the study, ED symptoms were measured using the self-administered online Eating Disorder Examination Questionnaire (EDE-Q) [21]. Further description of the EMKIE study can be found elsewhere [21–23].

### Ethics

This study was conducted in accordance with the Declaration of Helsinki [24]. Review and approval were given by the Ethics Committee of the Medical Faculty of Eberhard Karls University and the University Hospital of Tuebingen (#219/2018/BO1, #859/2021/BO2). All participants provided written informed consent.

### Measures

**Fig. 1:**
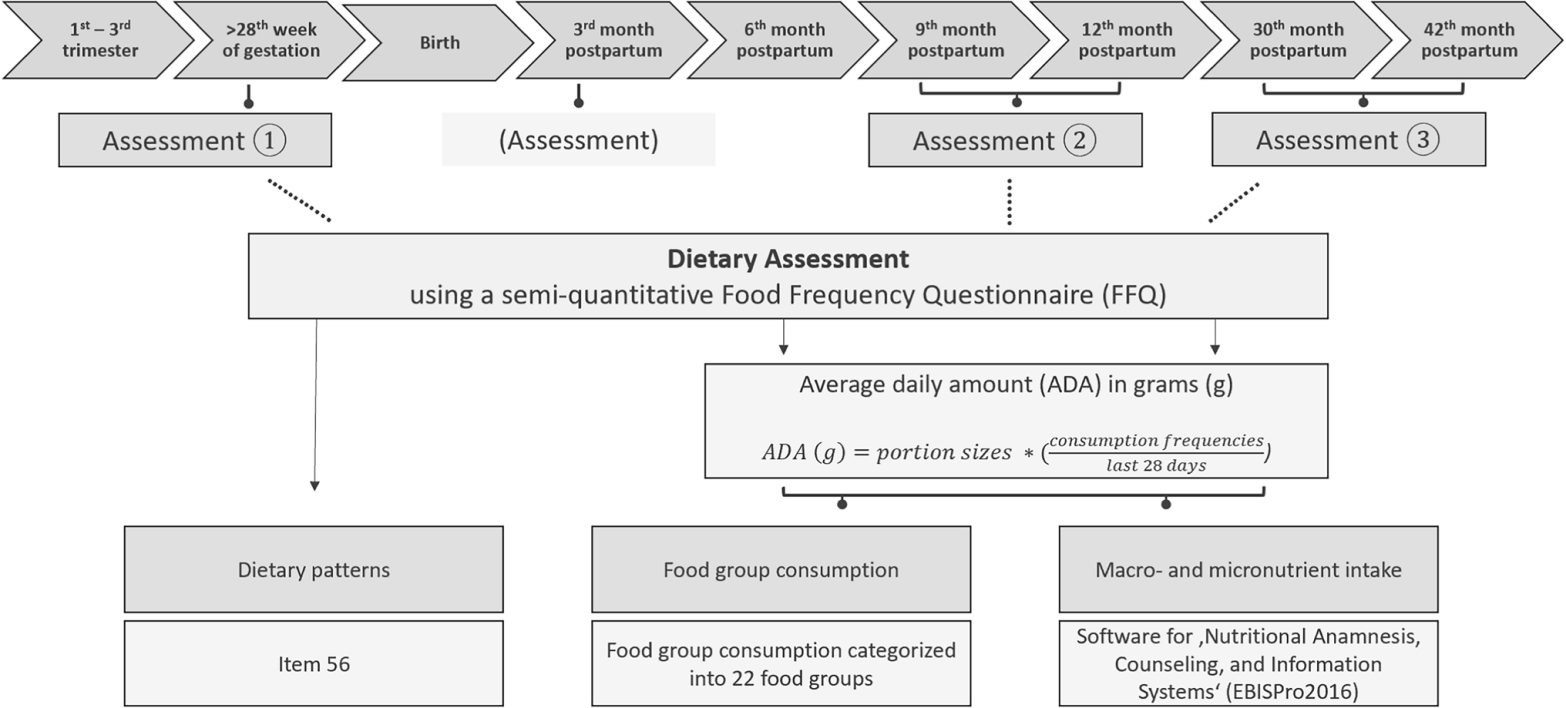
Study procedure and formula for average daily amounts

Dietary intake was assessed during pregnancy (third trimester, T1), 1 year postpartum (9–12 months, T2), and 3 years postpartum (30–42 months, T3) using a semi-quantitative food frequency questionnaire (FFQ) [25]. The FFQ used in this study was developed and validated within the German Health Interview and Examination Survey for Adults (DEGS-1) [25]. It refers to the diet of the preceding 28 days and comprises 53 items assessing the frequency and quantity of food consumption, supplemented by four additional items on food preparation (fats and oils used), dietary patterns (vegetarian, pescetarian, and vegan) and cooking habits. The FFQ included portion size images and written portion definitions for food items with less easily identifiable portion sizes (e.g., a small dessert bowl defined as 150 ml of strawberries). Data analysis was partly based on the average daily amounts in grams (g/day). For each participant, the average daily amount of each food at each assessment was calculated by dividing the consumption frequency over the last 28 days by 28, then multiplying the result by the portion size (Fig. 1). Questionnaire data collected at T1, T2, and T3 were analysed to determine dietary patterns, food group consumption, and macro- and micronutrient intake. An overview of the study procedure is shown in Fig. 1.

### Dietary patterns

Dietary patterns were assessed using item 56 of the FFQ, which consists of two questions, namely: (a) ‘*Do you usually eat a vegetarian diet?*’ and (b) ‘*Which of the following foods do you avoid? Multiple answers possible. (1) meat, poultry, and processed meat; (2) fish; (3) milk and dairy products; (4) eggs*’.

### Food group consumption

The 53 food items of the FFQ were grouped into twenty predefined food groups, which were adapted from the DEGS-1 study [26]. For each participant at each assessment, average daily amounts of all foods within a group were summed to derive total group-level intake.

### Macro- and micronutrient intake

Average daily amounts at each assessment were entered into the analysis software ‘*Nutritional Anamnesis, Counselling, and Information System’ (EBISPro, Version 2016)* [27]. Macro- and micronutrient intakes were then calculated based on the ‘*German Federal Food Code’* for each participant [28].

### Data analysis

Statistical analysis was performed using IBM SPSS Statistics software for Windows, Version 28.0. Normal distribution of the data was tested using Shapiro–Wilk test, box plots, and histograms. Group differences were reported using the unpaired *t*-test for metric normally distributed data. Metric non-normally distributed data were reported using the Mann–Whitney-U test. Categorical variables and nominal frequencies were tested for significance using the Chi-Square test or Fisher's exact test. Statistical changes over the course of the study were calculated using Friedman's two-factor analysis of variance and Cochran's Q test (for binary responses). Descriptive characteristics were reported as means (M), standard deviations (SD), confidence intervals (CI) or percentages (%). The level of significance was set at *α* = 0.05. The effect size *r* (*r*) for the Mann–Whitney-U test; the Phi-coefficient (*φ*) for the Chi-Square test (for 2 × 2 tables) and Fisher's exact test, Cohen's *d* (*d*) for the unpaired t-test, and Kendall's *W* (*w*) for the Friedman test were interpreted according to Cohen [29] as follows: small: *r, φ, w* = 0.10; *d* = 0.20, medium: *r, φ, w* = 0.30; *d* = 0.50, large: *r, φ, w* = 0.50; *d* = 0.80.

## Results

### Participant characteristics

The sample consisted of 57 women (ED: *n* = 24; HC: *n* = 33). Diagnoses within the ED group were AN (*n* = 16), BN (*n* = 4), BED (*n* = 1), and OSFED (*n* = 3), with 50.0% of the ED group in remission at T1 (mean remission duration = 4.5 ± 5.4 years). At T2, one participant dropped out, resulting in 56 participants (ED: *n* = 23, HC: *n* = 33). At T3, 13 participants did not complete the questionnaire, resulting in 43 participants (ED: *n* = 16, HC: *n* = 27). Maternal characteristics at T1 are presented in Table 1. There were no significant differences in body mass index (BMI) between the groups at T2 and T3 (T2: ED: M = 23.16 kg/m^2^, HC: M = 21.70 kg/m^2^, *p* = 0.918; T3: ED: M = 22.95 kg/m^2^, HC: M = 22.37 kg/m^2^, *p* = 0.693). Participants lost to follow-up at T3 did not differ significantly from completers with respect to age, BMI, ED diagnosis, ED symptom severity, or sociodemographic characteristics at baseline (all p > 0.05).

**Table 1:** Maternal characteristics at T1

Maternal characteristics (T1)	Total sample (*n* = 57)	ED (*n* = 24)	HC (*n* = 33)	*p*-value	Effect size
	*M* ± *SD*	*M* ± *SD*	*M* ± *SD*		
Age (years)	31.30 ± 4.18	31.83 ± 3.99	30.91 ± 4.33	0.415^a^	0.22^d^
BMI (kg/m^2^)	25.42 ± 4.14	25.78 ± 5.64	25.16 ± 2.66	0.734^b^	0.05^r^
Pre-pregnancy BMI (kg/m^2^)	22.14 ± 3.99	22.77 ± 5.40	21.68 ± 2.51	0.923^b^	0.01^r^
Gestational week	30.96 ± 2.81	30.79 ± 3.05	31.09 ± 2.66	0.442^b^	0.10^r^
Gestational weight gain (kg)	9.43 ± 3.87	8.67 ± 4.23	9.98 ± 3.56	0.221^a^	−0.34^d^
Nullipara (%)	68.40	66.70	69.70	0.919^c^	−0.05^φ^
Primipara (%)	21.10	20.80	21.20	0.919^c^	−0.05^φ^
Pluripara (%)	10.60	12.50	9.10	0.919^c^	−0.05^φ^
University degree (%)	73.70	66.70	78.80	0.368^d^	0.14^φ^
In a partnership (%)	96.50	91.70	100.00	0.253^c^	0.24^φ^
Family income > 4000 € (%)	56.10	50.00	60.60	0.582^c^	0.19^φ^

Data presented as means (M); standard deviations (SD) or percentages (%)

*BMI* body mass index, *ED* eating disorder group, *HC* healthy control group

^a^Student’s *t*-test

^b^Mann-Whitney-U test

^c^Fishers’s exact test

^d^Chi-Square test

### Eating disorder psychopathology

As previously reported by Throm et al. [23], eating disorder psychopathology differed significantly between groups across all assessments and increased significantly from pregnancy to 1 year postpartum, with a more pronounced rise in the ED group [23]. Three years postpartum, which was additionally included in the present analysis, group differences remained significant (*z* = −3.69; *p* < 0.001; *r* = 0.56). EDE-Q global scores continued to increase over time (total sample: *x*^2^(2) = 24.52; *p* < 0.001; *w* = 0.29), particularly pronounced in the ED group (*x*^*2*^(2) = 16.00; *p* < 0.001; *w* = 0.53). Bonferroni-adjusted post hoc comparisons showed a significant increase from T1 to T3, with no change between 1 and 3 years postpartum (total sample *p* < 0.001; ED: *p* = 0.001).

### Dietary patterns

Most participants reported an adherence to omnivorous diets (T1: 75.4%, T2: 73.2% T3: 74.4%). While alternative dietary patterns (vegetarian, pescetarian, and vegan) were observed, no significant group differences (all *p* > 0.05) or changes over time (*p* = 0.203) were found.

### Food groups

Several group differences in food group consumption emerged throughout the study (Table 2). During pregnancy, significantly more energy-dense foods such as pizza, sweets, sweet spreads, as well as meat and poultry were consumed by the HC group. One year postpartum, significantly higher intakes of animal-based foods, convenience foods, and alcoholic beverages were observed among the HC group. Three years postpartum, the pattern of higher intakes of energy-dense, animal-based and convenience foods persisted.

**Table 2:** Differences in food group consumption over the course of study

Food groups (g/day)	T1				T2				T3			
	Third trimester				9–12 months postpartum				30–40 months postpartum			
	Mean [95% CI]		*p-value*	Effect size	Mean [95% CI]		*p-*value	Effect size	Mean [95% CI]		*p*-value	Effect size
	*ED (n* = *24)*	*HC (n* = *33)*			*ED (n* = *23)*	*HC (n* = *33)*			*ED (n* = *16)*	*HC (n* = *27)*		
Milk and dairy products	444.1 [260.5; 627.7]	362.5 [273.0; 451.9]	0.936^U^	−0.01^*r*^	400.9 [217.1; 584.7]	390.6 [294.1; 487.1]	0.771^U^	−0.04^*r*^	377.6 [154.5; 600.7]	302.9 [222.0; 383.9]	0.885^U^	−0.03^*r*^
Tea	319.8 [51.7; 587.9]	391.6 [126.1; 657.2]	0.335^U^	−0.13^*r*^	407.0 [125.6; 688.4]	224.4 [129.1; 319.7]	0.587^U^	−0.07^*r*^	287.9 [15.8; 559.9]	212.1 [130.6; 293.6]	0.407^U^	−0.13^*r*^
Coffee	126.8 [41.1; 212.4]	130.8 [75.2; 186.5]	0.403^U^	−0.11^*r*^	263.7 [170.9; 356.5]	311.5 [194.4; 428.7]	0.852^U^	−0.03^*r*^	353.2 [91.1; 615.3]	232.4 [159.3; 305.6]	0.789^U^	−0.04^*r*^
Non–alcoholic beverages	253.8 [90.5; 417.1]	264.9 [95.5; 434.2]	0.599^U^	−0.07^*r*^	273.3 [−61.0; 607.7]	190.2 [67.8; 312.7]	0.221^U^	−0.16^*r*^	173.5 [47.8; 299.3]	168.3 [71.8; 264.8]	0.609^U^	−0.08^*r*^
Water	2929.2 [2200.0; 3658.4]	2842.4 [2230.8; 3454.1]	0.973^U^	−0.00^*r*^	2163.0 [1459.7; 2866.4]	2366.7 [1805.3; 2928.0]	0.542^U^	−0.08^*r*^	1841.9 [964.0; 2719.8]	2106.9 [1437.9; 2775.9]	0.455^U^	−0.12^*r*^
Alcoholic beverages	0.0	0.5 [−0.4; 1.4]	0.224^U^	−0.16^*r*^	0.5 [−0.5; 1.5]	21.3 [5.0; 37.7]	**0.002**^**U**^	−0.41^*r*^	8.5 [−0.3; 17.2]	26.2 [4.1; 48.4]	0.295^U^	−0.16^*r*^
Bread and cereals	100.2 [75.5; 124.8]	101.9 [84.7; 119.1]	0.903^t(u)^	−0.03^*d*^	128.3 [88.9; 167.7]	115.9 [96.9; 134.9]	0.524^t(u)^	−0.17^*d*^	135.7 [76.4; 195.0]	115.1 [84.6; 145.6]	0.627^U^	−0.08^*r*^
Butter and margarine	3.2 [1.4; 5.1]	5.5 [3.0; 7.9]	0.152^U^	−0.19^*r*^	4.1 [1.4; 6.8]	7.9 [4.5; 11.3]	**0.006**^**U**^	−0.37^*r*^	2.7 [−0.2; 5.7]	7.9 [4.8; 11.0]	**0.001**^**U**^	−0.50^*r*^
Sweet spreads	6.6 [2.8; 10.4]	10.8 [7.1; 14.5]	**0.041**^**U**^	−0.27^*r*^	6.2 [1.4; 10.9]	9.0 [5.5; 12.6]	0.071^U^	−0.24^*r*^	3.9 [0.3; 7.4]	7.5 [4.6; 10.3]	**0.009**^**U**^	−0.40^*r*^
Eggs	9.1 [5.2; 13.0]	14.5 [8.8; 20.3]	0.238^U^	−0.16^*r*^	9.4 [5.4; 13.3]	15.1 [10.6; 19.6]	0.073^U^	−0.24^*r*^	15.8 [4.9; 26.7]	17.9 [11.4; 24.5]	0.403^U^	−0.13^*r*^
Meat and poultry	21.2 [6.8; 35.6]	35.2 [25.0; 45.4]	**0.006**^**U**^	−0.36^*r*^	17.2 [7.5; 26.9]	37.1 [22.8; 51.4]	**0.017**^**U**^	−0.32^*r*^	14.9 [3.6; 26.3]	39.1 [27.5; 50.7]	**0.006**^**U**^	−0.43^*r*^
Fast food	9.7 [4.7; 14.7]	14.8 [9.4; 20.2]	0.165^U^	−0.18^*r*^	5.9 [1.1; 10.6]	12.9 [7.1; 18.8]	**0.027**^**U**^	−0.30^*r*^	6.7 [1.2; 12.1]	23.9 [12.4; 35.5]	**0.011**^**U**^	−0.40^*r*^
Cold cuts	5.7 [2.0; 9.4]	7.7 [4.4; 11.1]	0.135^U^	−0.20^*r*^	4.8 [1.9; 7.8]	13.0 [5.8; 20.2]	**0.028**^**U**^	−0.29^*r*^	5.8 [−0.8; 12.5]	7.7 [4.2; 11.2]	**0.042**^**U**^	−0.31^*r*^
Fish	7.6 [4.4; 10.9]	12.2 [8.6; 15.8]	0.056^U^	−0.25^*r*^	8.2 [2.1; 14.2]	16.1 [10.0; 22.2]	**0.008**^**U**^	−0.36^*r*^	14.9 [−1.6; 31.4]	51.4 [−30.5; 133.3]	0.243^U^	−0.18^*r*^
Fruit	393.6 [255.7; 531.6]	352.9 [244.5; 461.3]	0.884^U^	−0.02^*r*^	292.8 [212.5; 373.1]	223.0 [167.6; 278.5]	0.119^U^	−0.21^*r*^	453.0 [239.9; 666.2]	248.9 [183.8; 314.1]	0.221^U^	−0.19^*r*^
Vegetables and legumes	484.7 [296.0; 673.4]	337.7 [233.7; 441.6]	0.599^U^	−0.09^*r*^	395.4 [237.6; 553.2]	305.6 [223.1; 388.1]	0.739^U^	−0.05^*r*^	709.8 [260.6; 1159.1]	285.2 [210.5; 359.9]	0.185^U^	−0.21^*r*^
Rice, pasta, and potatoes	111.3 [80.2; 142.4]	137.0 [106.7; 167.3]	0.286^U^	−0.14^*r*^	129.1 [91.2; 167.0]	156.1 [133.7; 178.5]	0.230^U^	−0.16^*r*^	116.6 [78.5; 154.7]	138.7 [112.4; 165.1]	0.341^t(u)^	−0.33^*d*^
Pizza	11.7 [6.7; 16.7]	31.2 [20.4; 42.0]	**0.001**^**U**^	−0.49^*r*^	13.7 [7.7; 19.8]	21.2 [14.7; 27.7]	0.163^U^	−0.19^*r*^	11.7 [2.2; 21.2]	32.3 [18.2; 46.4]	**0.005**^**U**^	−0.43^*r*^
Sweets	43.4 [26.2; 60.6]	65.8 [49.4; 82.2]	**0.021**^**U**^	−0.31^*r*^	81.9 [7.3; 156.5]	76.7 [55.7; 97.7]	0.057^U^	−0.25^*r*^	61.3 [23.6; 98.9]	74.8 [44.7; 104.8]	0.503^U^	−0.10^*r*^
Snacks	12.1 [5.2; 19,1]	9.4 [5.6; 13.1]	0.686^U^	−0.04^*r*^	10.9 [4.8; 17.0]	8.5 [5.5; 11.5]	0.855^U^	−0.26^*r*^	14.1 [5.0; 23.3]	11.8 [7.2; 16.4]	0.713^U^	−0.06^*r*^

Data presented as means (M) and 95% confidence intervals (95% CI). Significant differences (*p *< 0.05) are shown in bold. *U* Mann–Whitney–U test, *t(u)* Students t test, *r* effect size *r*, *d* Cohen’s *d*

### Macro- and micronutrients

**Fig. 2:**
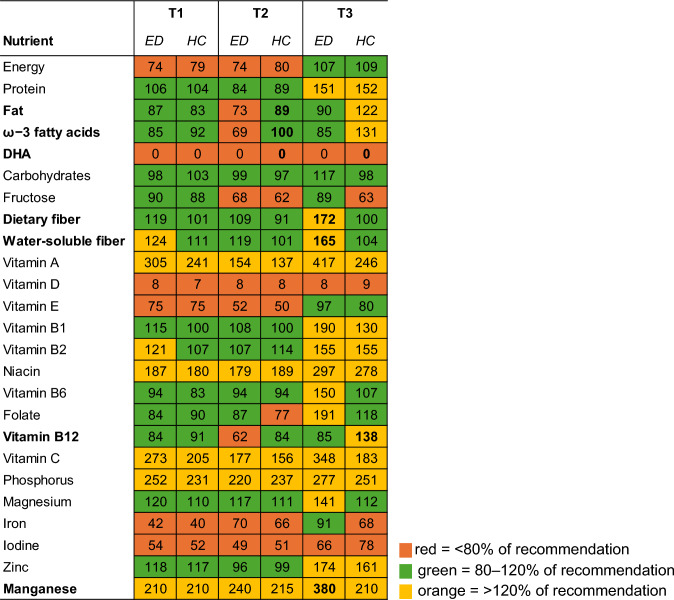
Percentage of recommended nutrient intake throughout the study. Reference values according to pregnant women (T1), breastfeeding women (T2), and women between 25 and 51 years of age (T3). Bold numbers indicate group with significantly (*p* < 0.05) higher intakes

No significant group differences in nutrient intakes were observed at T1. At T2, the HC group showed significantly higher intakes of total fat, including saturated (SFA), monounsaturated (MFA), and polyunsaturated fatty acids (PUFA), as well as omega-3 fatty acids (ω − 3) and docosahexaenoic acid (DHA), compared to the ED group (fat: *z* = 2.21; *p* = 0.027; *r* = 0.29; SFA: *z* = 2.09; *p* = 0.037; *r* = 0.27; MUFA: *z* = 2.31; *p* = 0.021;* r* = 0.30; PUFA: *z* = 2.32; *p* = 0.021; *r* = 0.30; ω − 3: *z* = 3.07; *p* = 0.002; *r* = 0.38; DHA: *z* = 2.81; *p* = 0.005; *r* = 0.35). At T3, the ED group had significantly higher intakes of dietary fiber and water-soluble fiber (*z* = −2.09; *p* = 0.037;* r* = −0.32; *z* = −2.01; *p* = 0.044;* r* = 0.31), as well as manganese (*z* = −2.31; *p* = 0.021;* r* = 0.35), all exceeding recommended levels. In contrast, the HC group showed significantly higher vitamin B12 intakes (*z* = −2.16; *p* = 0.031;* r* = 0.33), also above recommendations. Several nutrient recommendations were not met in either group across the study (Fig. 2).

## Discussion

In this longitudinal study, dietary intake among women with and without ED history during pregnancy and the postpartum period was examined. Most participants reported an adherence to omnivorous diets and overall nutrient intake was largely comparable between groups. Distinct differences were observed at food group level: women with ED history reported a lower consumption of energy-dense, convenience, and animal-based foods, which was partially reflected in micronutrient analysis.

### Dietary patterns

No significant group differences or changes over time were observed. However, previous findings suggest that women with ED history were more likely to follow vegetarian diets and consume less meat [1, 17]. Paslakis et al. [30] additionally reported that female gender, younger age, lower BMI, higher depression scores and elevated ED symptoms were significantly associated with alternative dietary patterns [30]. This may be because of heightened body-related concerns, health-conscious motivations or perceived dietary control. Ethical and ecological considerations may also determine dietary choices [31].

### Food groups

Women with a history of ED consistently consumed fewer energy-dense, sugary, fatty and animal-based foods than healthy controls, a pattern aligning with previous findings of greater health consciousness and higher diet quality in this population during pregnancy [1, 17]. This pattern may reflect pregnancy and the postpartum period as phases of heightened dietary awareness in women with ED history, potentially accompanied by a stronger motivation to promote perceived optimal outcomes for maternal and child health. Given their often substantial nutritional knowledge [10–12], women with ED history may consciously select foods perceived as particularly health-promoting. At the same time, the avoidance of specific food groups does not necessarily indicate a more protective nutritional profile. Restriction of energy-dense or animal-based products may increase the risk of insufficient intake of certain nutrients, suggesting that the observed dietary behavior may reflect both adaptive health awareness and potentially unbalanced dietary patterns. Alternatively, the reduction of energy-dense foods may also be driven by concerns about (gestational) weight gain and serve as a compensatory strategy. In this context, the longitudinal increase in ED psychopathology observed in the cohort may indicate that underlying cognitive patterns of disordered eating continue to shape food-related decision-making, despite apparent behavioral normalization. Notably, differences in food group consumption were already present during pregnancy, suggesting that these dietary patterns may reflect more enduring characteristics associated with ED history rather than changes solely driven by symptom severity over time. However, reporting bias and the high educational level of the cohort may also have contributed to this pattern. One year postpartum, alcohol intake was higher in the HC, although overall consumption levels remained low. As even low alcohol intake carries risks, this warrants caution [32].

### Macro- and micronutrients

During pregnancy, no group differences in nutrient intake were observed, consistent with previous findings showing comparable intakes in women with and without ED history [2, 17]. Both groups failed to meet recommended intakes for several critical nutrients across pregnancy and lactation, underscoring the need for targeted supplementation. One year postpartum, women with ED history reported lower fat intakes, aligning with prior studies [1, 17, 18]. Reduced fat consumption may reflect an avoidance of ‘fattening’ foods [33, 34], which is notable, given the importance of PUFAs for maternal health and child neurodevelopment [4]. Three years postpartum, higher dietary fiber and manganese intakes as well as lower vitamin B12 intake within the ED group were consistent with the observed reduced meat consumption and health-conscious dietary pattern. While this is generally indicative of healthy eating, this pattern may also be associated with potential nutritional risks, e.g., insufficient vitamin B12 intake in the context of meat-reduced diets [35]. These findings indicate that health-oriented dietary patterns do not necessarily equate to optimal nutrient adequacy, particularly when specific food groups are selectively reduced. This may be relevant in women with ED history, where food avoidance may coexist with motivations for healthy eating. Nevertheless, nutrient-level estimates should be interpreted cautiously, as they are more susceptible to measurement errors than food group analyses.

### Strength and limits

This study enabled the evaluation of long-term dietary intake by extending follow-up to 3 years, in contrast to previous research limited to pregnancy [1, 17, 18]. ED diagnoses were established at study inclusion using the EDE-I, allowing classification into ED and HC groups. Repeated assessment of psychopathology provided insight into symptom severity over time. Although BMI adjusted in both groups after pregnancy, rising EDE-Q scores indicated heightened vulnerability within this cohort. Dropout was minimal until T2 but increased at T3 following the retrospective addition of this assessment, which limited re-contact with participants, followed by missing data for 13 participants. Furthermore, given the limited sample size, this study was not powered to detect small effects. The analyses should therefore be considered exploratory, and the findings warrant cautious interpretation and confirmation in larger studies. Dietary intake was assessed using an online FFQ, a widely used method in European cohort studies [36], although prone to systematic errors such as recall bias, portion size misestimation, and social desirability, with limited accuracy for absolute nutrient estimates [37, 38]. Portion size perception may be particularly affected in women with EDs [39, 40]. However, the use of visual aids and written portion definitions within the FFQ may have supported portion size estimation, which may be particularly relevant in this cohort. As dietary behaviors and nutritional risks may vary across ED subtypes and illness stages, the absence of subgroup analyses represents an important limitation and should be considered when interpreting the findings. Participants were recruited within the Tuebingen region (Baden-Württemberg, Germany), resulting in a geographically limited sample. Selection bias must also be considered, as participants were predominantly well educated and may represent individuals with a higher interest in health-related research participation, limiting generalizability of these findings to more heterogeneous populations. Finally, the absence of validation measures (e.g., biomarkers) restricts the precision of nutrient estimations.

## Implications

These findings have important clinical implications, as differences in food group consumption, together with increasing ED psychopathology, may point to a period of heightened psychosocial and nutritional vulnerability in women with ED history during the postpartum period. Greater awareness among obstetric, mental health, and nutritional professionals therefore appears warranted. Counselling approaches may benefit from a sensitive and individualized approach that addresses both nutritional adequacy and potential persistence of restrictive tendencies. Further large-scale longitudinal studies are needed to further clarify the complex relationship between dietary intake, ED symptom trajectories, and potential nutritional risks across pregnancy and the postpartum period.

## What is already known on this subject?

The few existing studies indicate that women with ED history may show distinct dietary patterns during pregnancy. Although ED symptoms are often reduced during pregnancy, they might intensify after birth. Despite this well-documented postpartum increase in psychopathology, research has scarcely assessed dietary behavior during the vulnerable postpartum period.

## What this study adds?

This study provides the first longitudinal assessment of dietary intake from pregnancy to 3 years postpartum in women with ED history, identifying persistent differences in food group consumption and possible related potential nutrient vulnerabilities.

## Conclusion

Most women in the total sample followed an omnivorous diet. Importantly, nutrient intakes were comparable between groups during pregnancy, which is encouraging given the heightened vulnerability during this period. The most pronounced group differences emerged in food group consumption, with women with ED history displaying more health-conscious patterns. These findings may reflect a dual phenomenon: an increased health awareness and a preference for nutritious diets, alongside potentially residual restrictive tendencies, which may relate to the increase in ED psychopathology observed in this cohort. Taken together, these findings highlight the postpartum period as a psychosocially and nutritionally vulnerable phase and underline the need for targeted support to ensure adequate nutrition in women with ED history.

## Data Availability

Data can be requested from the corresponding author.
